# Biochemical detection of fatal hypothermia and hyperthermia in affected rat hypothalamus tissues by Fourier transform infrared spectroscopy

**DOI:** 10.1042/BSR20181633

**Published:** 2019-03-15

**Authors:** Hancheng Lin, Kaifei Deng, Ji Zhang, Lei Wang, Zhong Zhang, Yiwen Luo, Qiran Sun, Zhengdong Li, Yijiu Chen, Zhenyuan Wang, Ping Huang

**Affiliations:** 1 Shanghai Key Laboratory of Forensic Medicine, Shanghai Forensic Service Platform, Academy of Forensic Science, Shanghai 200063, China; 2 Department of Forensic Pathology, Xi’an Jiaotong University, Xi’an 710061, China

**Keywords:** Chemometrics, Fatal hypothermia, Fatal hyperthermia, Fourier transform infrared spectroscopy, Hypothalamus, Postmortem diagnosis

## Abstract

It is difficult to determinate the cause of death from exposure to fatal hypothermia and hyperthermia in forensic casework. Here, we present a state-of-the-art study that employs Fourier-transform infrared (FTIR) spectroscopy to investigate the hypothalamus tissues of fatal hypothermic, fatal hyperthermic and normothermic rats to determine forensically significant biomarkers related to fatal hypothermia and hyperthermia. Our results revealed that the spectral variations in the lipid, protein, carbohydrate and nucleic acid components are highly different for hypothalamuses after exposure to fatal hypothermic, fatal hyperthermic and normothermic conditions. In comparison with the normothermia group, the fatal hypothermia and hyperthermia groups contained higher total lipid amounts but were lower in unsaturated lipids. Additionally, their cell membranes were found to have less motional freedom. Among these three groups, the fatal hyperthermia group contained the lowest total proteins and carbohydrates and the highest aggregated and dysfunctional proteins, while the fatal hypothermia group contained the highest level of nucleic acids. In conclusion, this study demonstrates that FTIR spectroscopy has the potential to become a reliable method for the biochemical characterization of fatal hypothermia and hyperthermia hypothalamus tissues, and this could be used as a postmortem diagnostic feature in fatal hypothermia and hyperthermia deaths.

## Introduction

Thermoregulation, as one important aspect of human homeostasis, keeps the body temperature of the organism within certain boundaries [1]. The prolonged exposure to extreme ambient temperature (fatal hypothermia and hyperthermia) can induce the breakdown of the internal thermoregulatory control and finally, especially for the very young and the elderly, lead to death [2,3]. The identification of hypothermia and hyperthermia as the cause of death is usually difficult in forensic casework because of unspecific, irregular or even negative macroscopic and microscopic findings [2–7]. In order to better understand the pathophysiology of fatal hypothermia and hyperthermia, numerous postmortem biochemical investigations of biochemical markers such as electrolytes, hormones, blood proteins, enzymes and neurotransmitters, in the blood and other biological fluids, have been performed in the last several decades [2–4,8–16]. However, the practical applications of these biochemical investigation results need to be confirmed through targeted studies on larger sample groups of hypothermia/hyperthermia-related deaths.

The hypothalamus is the main seat of thermoregulation, and its metabolic and functional activity changes as the core temperature of the body changes [17]. Therefore, it is reasonable to expect that a good knowledge of the biochemical hypothalamus response to fatal hypothermic/hyperthermic stress would be beneficial for the determination of fatal hypothermia/hyperthermia. However, only a few of the published studies on hypothalamus tissue discuss the changes of specific immunohistochemistry-specific markers, like catecholamines [18] and chromogranin A [19], in response to fatal hypothermia/hyperthermia. The overall biochemical response of the hypothalamus is still unclear. It would be highly advantageous to implement a simple and rapid method for the acquisition of total biomolecular composition and the variations, to be able to analyze hypothalamus tissue for forensic cases.

Fourier-transform infrared (FTIR) spectroscopy is a qualitative and quantitative analytical technique that is commonly used in the fields of biomedical science, materials science and medicine [20–22]. The principle of infrared spectroscopy is to utilize an infrared source-induced molecular motion to generate a signature fingerprint of chemical components in the form of spectrum [23]. The increased improvement in both instrumentation (such as surface enhanced infrared spectroscopy, quantum cascade laser infrared spectroscopy and atomic force microscopy infrared spectroscopy) [24–26] and chemometric methods (such as artificial neural network, support vector machine, random forest algorithm and linear discriminant analysis) [27] has seen infrared spectroscopy become a powerful technique for analyzing biological samples, like cells, tissues and biological fluids. The most commonly used infrared region for biological applications is the mid-IR region (4000–400 cm^−1^), which includes the 3100–2800 cm^−1^ region, mainly representative of lipids, and the so-called fingerprint region (1800–900 cm^−1^), mainly representative of amide I/II, lipids, carbohydrates and nucleic acids [28]. The greatest benefit of FTIR technique lies in its highly sensitive acquisition of the whole ‘-omics’ of a biological sample, which enables the detection of molecular changes that may reflect the onset and progression of a disease, thus enabling disease diagnosis and monitoring [29]. Up to now, this technique has been widely used in the medical field for cancer diagnoses, such as skin [30], breast [31,32], cervical [33], colon [34] and kidney tumors [35], detections of antibiotics’ resistant *Escherichia coli* bacteria [36], human papillomavirus [37] and malaria infected red blood cells [38], and monitoring of tendinopathy [39] and chronic venous leg ulcer exudates [40].

The potential of FTIR techniques is also highlighted in the research of neurodegenerative diseases, like Alzheimer’s disease [41–44], Parkinson’s disease [45–47] and multiple sclerosis [48], since FTIR spectroscopy is sensitive to protein aggregation, which is regarded as the hallmark of neurodegenerative diseases in an emerging concept in the field of central nervous system diseases [49]. Additionally, there is abundant literature reporting the applications of FTIR spectroscopy to other neurological diseases such as, cerebral malaria [50], ischemic stroke [51–54], hemorrhagic stroke [55] and epilepsy [56,57], in human and animal models. These published studies demonstrate neurological disease-related biochemical alternations such as protein misfolding and aggregation, lipid oxidation, abnormal carbohydrate metabolism and DNA/RNA unusual expression, all monitored by FTIR spectroscopy.

Given the powerful capacity of FTIR spectroscopy for detecting specific spectral biomarkers for brain tissues with various pathological conditions, we established a state-of-the-art study that employed the FTIR technique with a combination of chemometric methods to investigate the biochemical changes of hypothalamus tissues in response to fatal hypothermia, fatal hyperthermia and normothermia in rat models. Hypothalamus not only regulates body temperature, but also controls hunger, important aspects of parenting and attachment behaviors, thirst, fatigue, sleep and circadian rhythms [58]. It’s reasonable to believe that except the temperature factor, changes of the other factors could also lead to alternations of hypothalamus functions and biochemical properties. To eliminate the effects of these factors on the biochemical properties of hypothalamus tissue, a targeted feeding programme for experimental rats was designed in this study. Ultimately, it is guaranteed that the changes of biochemical properties of hypothalamus are mainly the result of pathophysiological processes induced by extreme temperature and(or) the result of the direct physical effect of extreme temperature on the hypothalamus tissue. The aim of our study is to identify differences and similarities in the proteomic, lipidemic, genomic and metabolic components of the fatal hypothermic, fatal hyperthermic and normothermic hypothalamus tissues. The comparison of these could give new insights into the pathophysiological process of the hypothalamus in response to fatal hypothermia and hyperthermia stress. Furthermore, the specific spectrochemical markers that determined by chemometric methods may serve us to develop new method for postmortem diagnosis of fatal hypothermia and hyperthermia.

## Materials and methods

### Animal preparation

The study was conducted in strict accordance with the recommendations in the Guide for the Care and Use of Laboratory Animals of Xi’an Jiaotong University. The protocol was approved by the Committee on the Ethics of Animal Experiments of Xi’an Jiaotong University. Every effort was made to minimize animal suffering. Forty-seven male Sprague–Dawley rats weighing 260–300 g (provided by the Animal Centre of Xi’an Jiaotong University) were used for the experiment. The rat models were established as described previously [59,60]. Briefly, the rats were kept for one week in stainless steel cages at 23 ± 2°C until physical conditions were stabilized in a 12-h light/dark environment. Food and water were supplied *ad libitum*. Then, the rats were anaesthetized with an intraperitoneal injection of pentobarbital sodium (50 mg/kg) and randomly divided into three groups. In the fatal hyperthermia group (*n*=17), the rats were exposed to an ambient temperature of 43°C in a temperature-controlled chamber with relative humidity of 60% until death (average death time, 80 min). In the fatal hypothermia group (*n*=17), the dorsal and abdominal hair of the rats were shaved and thereafter the area was immersed in ethanol (96%) for about 10 s. After ethanol exposure, the rats were placed in a cold room at 4°C until death (average death time, 120 min). In the control group (*n*=13), rats were humanely sacrificed through decapitation. When each rat was confirmed dead, its brain was removed rapidly, then snap-frozen with liquid nitrogen and stored at −80°C until the FTIR experiments. Just before the experiment, a brain sample was taken from the freezer and two adjacent 10-μm-thick coronal sections of the brain were cut with a cryo-microtome at −18°C. The details about the selection of specific brain coronal sections for infrared measurement are presented in Supplementary Figure S1. One section was melted onto a CaF_2_ substrate, and then air-dried for a period of 2 min for immediate FTIR imaging analysis. The other section was mounted on a conventional glass slide and stained with hematoxylin&eosin (H&E).

### FTIR spectroscopic analysis

FTIR spectroscopic images were collected using a Nicolet iN10 MX infrared microscope (Thermo Fisher Scientific, Waltham, MA, USA) equipped with a liquid nitrogen cooled 16-element mercury-cadmium-telluride linear array detector. The OMNIC Picta software 9.0 (Thermo Fisher Scientific, Waltham, MA, USA) was used for instrument control and spectral data acquisition. In this study, the spatial and spectral resolutions were set to 25 × 25 μm^2^ and 4 cm^−1^. Spectra were acquired with the co-addition of 32 scans over the range 4000−900 cm^−1^. A background image was collected from a blank substrate before the collection of each sample image. FTIR spectroscopic images of samples mounted on CaF_2_ slides were collected in transmission mode. In these conditions, approximately 4 min were needed to collect an infrared image containing 160 spectra and corresponding to a typical hypothalamus area of 250 × 400 μm^2^. It should be mentioned that before each measurement, the specific area of hypothalamus was confirmed by a professional pathologist. A total of 47 images (an image per sample) were finally recorded. A row spectral data set containing 7520 spectra (2720 for the fatal hypothermia group, 2720 for the fatal hyperthermia group and 2080 for the normothermia group) were gathered from these 47 images and then subjected to data pre-processing.

### Spectral data pre-processing and analysis

First, a 4-point baseline, passing by 3100, 2800, 1800 and 899 cm^−1^, was subtracted from each spectrum. Subsequently, the spectral dataset was subjected to extended multiplicative signal correction (EMSC) [61] to normalize the spectra and remove scattering effects. Finally, the dimensionalities of the spectral dataset were reduced to the 3100−2800 and 1800−900 cm^−1^, two specific regions dominated by the spectral features of biochemical components such as lipids, proteins, carbohydrates and nucleic acids. The pre-processed spectra were then subjected to principle component analysis (PCA) and random forest (RF). PCA is a classic unsupervised multivariate algorithm that can be used to categorize the samples based on the differences between the spectra [62]. PCA offers two important outcomes: one is PC-scores, which provides a way to understand and visualize the distribution of complex spectral datasets; the other is PC-loadings, which helps us identify new meaningful spectral variables underlying the distribution. RF is a supervised classification method whose one parameter, Mean Decrease in Gini Coefficient, could reveal important distinguishing wavenumbers [63]. PCA and RF were both implemented in this study to mine specific spectral features responsible for the discrimination of fatal hypothermic, fatal hyperthermic and normothermic hypothalamus tissues.

The pre-processing of the raw spectral dataset was performed using Unscrambler 10.3X (Camosoftware, Oslo, Norway). PCA was performed using the Statistics Toolbox built into MATLAB 2014a (MathWorks Inc., Natick, MA, USA). RF analysis was conducted using the ‘RandomForest’ Package (version 4.6-12) implemented in R 3.4.1.

### Statistical analysis

Normality of data was not guaranteed, so nonparametric methods of statistical analysis were adopted in this study. All data are presented as medians together with interquartile ranges. The statistical significance of differences between the analyzed groups was assessed by Kruskal–Wallis test. Nemenyi test was applied for multiple comparisons. A probability value of < 0.05 was considered significant. The statistical analysis was performed using the Statistics Toolbox built into MATLAB 2014a (MathWorks Inc., Natick, MA, USA).

## Results and discussion

### Histopathological analysis

**Figure 1:**
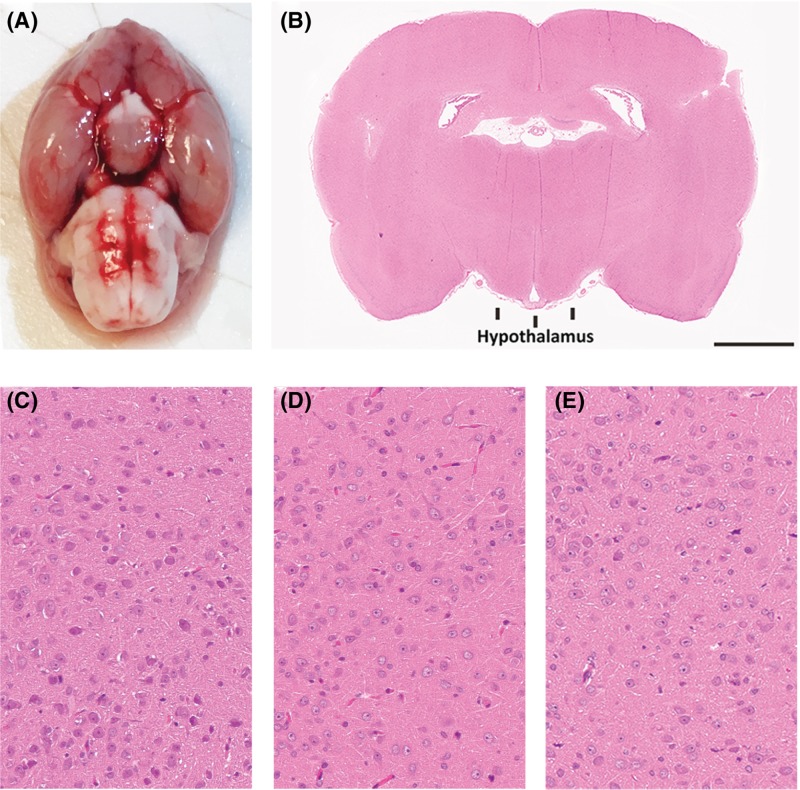
Histopathological results of the rat hypothalamus tissues (**A**) Normothermic rat brain annotated with an oval indicating the hypothalamus position; scale bar represents 2.5 mm. (**B**) Whole slide scan of an H&E stained normothermic brain tissue section annotated with a square indicating the IR measurement area of hypothalamus tissue. H&E staining results of the fatal hypothermic (**C**), hyperthermic (**D**) and normothermic (**E**) hypothalamus tissues; Scale bar represents 50 μm.

Histopathological analysis is a conventional procedure for forensic post-mortem examination. Generally, clear histopathological findings can help the examiner determine the antemortem pathophysiological state of the tissues [64]. In this study, to examine histopathological changes, the three groups of hypothalamus tissues were stained with H&E. An edematous and congestive characteristic of the hypothalamus was generally observed in the fatal hypothermic and hyperthermic samples (Figure 1). However, these findings are regarded as nonspecific in forensic pathology because similar changes can be observed in other cases with different causes of death.

### FTIR difference spectra analysis

**Figure 2:**
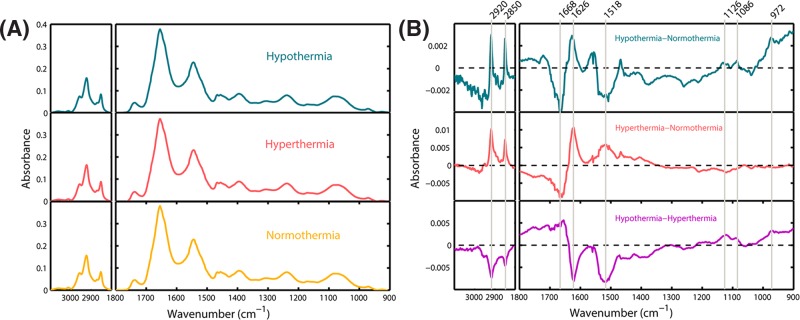
Results of FTIR spectral analysis (**A**) EMSC normalized and averaged IR spectra of fatal hypothermic, fatal hyperthermic and normothermic hypothalamus tissues. (**B**) IR difference spectra (i.e. hypothermia–normothermia, hyperthermia−normothermia and hypothermia−hyperthermia).

The EMSC normalized and averaged IR spectra of the three groups of hypothalamus tissue are displayed in Figure 2A. However, these three averaged spectra seem to be very similar at first glance. Therefore, in our next step, differential spectrum analysis was applied to capture the subtle spectral alterations. Figure 2B displays the new spectra calculated from the mean absorbance spectra shown in Figure 2A. The positive and negative signals representing absorbance differences are noticed mainly in the lipid region (3100−2800 cm^−1^), protein amide I and II bands (1700−1500 cm^−1^) and carbohydrate and nucleic acid region (1200−900 cm^−1^), indicative of the proteomic, lipidic, genomic and metabolic variations among the fatal hypothermic, fatal hyperthermic and normothermic hypothalamus tissues.

When the averaged absorbance spectrum of the normothermia group was subtracted from the fatal hypothermia and hyperthermia groups, their resultant difference spectra show quite similar band shapes in the lipid (3100−2800 cm^−1^) and protein amide I (1700−1600 cm^−1^) regions. In comparison with the normothermia group, the absorbance value at around 2850 and 2920 cm^−1^, attributed to the CH_2_ symmetric and asymmetric stretching mode of the fatty acids [65], increases in both the fatal hypothermia and hyperthermia groups. The observation of the negative peak at 1668 cm^−1^ (assigned as β-turn structures) [66] and the positive peak at 1626 cm^−1^ (assigned as β-sheet structures) [65] clearly demonstrates alterations of the secondary structures of regular proteins. However, the band shapes below 1530 cm^−1^ in the resultant difference spectra are quite different. The absorbance value at around 1518 cm^−1^, arising from tyrosine side chains [67], decreases in the case of the fatal hypothermia group but increases in the case of the fatal hyperthermia group. This finding suggests a different concentration of tyrosine-rich proteins in the fatal hypothermia and hyperthermia groups. Additionally, two positive peaks around 1086 and 972 cm^−1^ were detected for fatal hypothermia group; the 1086 cm^−1^ peak arises from symmetric PO^2−^ stretching of phosphodiester groups that could be found in DNA, RNA and phospholipids [65] and 972 cm^−1^ is attributed to C-C/C-O stretching of deoxyribose-ribose vibration of DNA [68,69]. These two positive peaks taken together suggest the existence of a higher amount of nucleic acids in the fatal hypothermia group in comparison with the normothermia group. In addition, another prominent IR peak that should be highlighted is the 1126 cm^−1^, an important spectral marker for lactate [51]. The increase and decrease for the fatal hypothermia and hyperthermia spectra respectively, in comparison with the normothermia spectrum, are indicative of different lactate concentrations in fatal hypothermic and hyperthermic hypothalamus tissues.

### Principle component analysis

**Figure 3:**
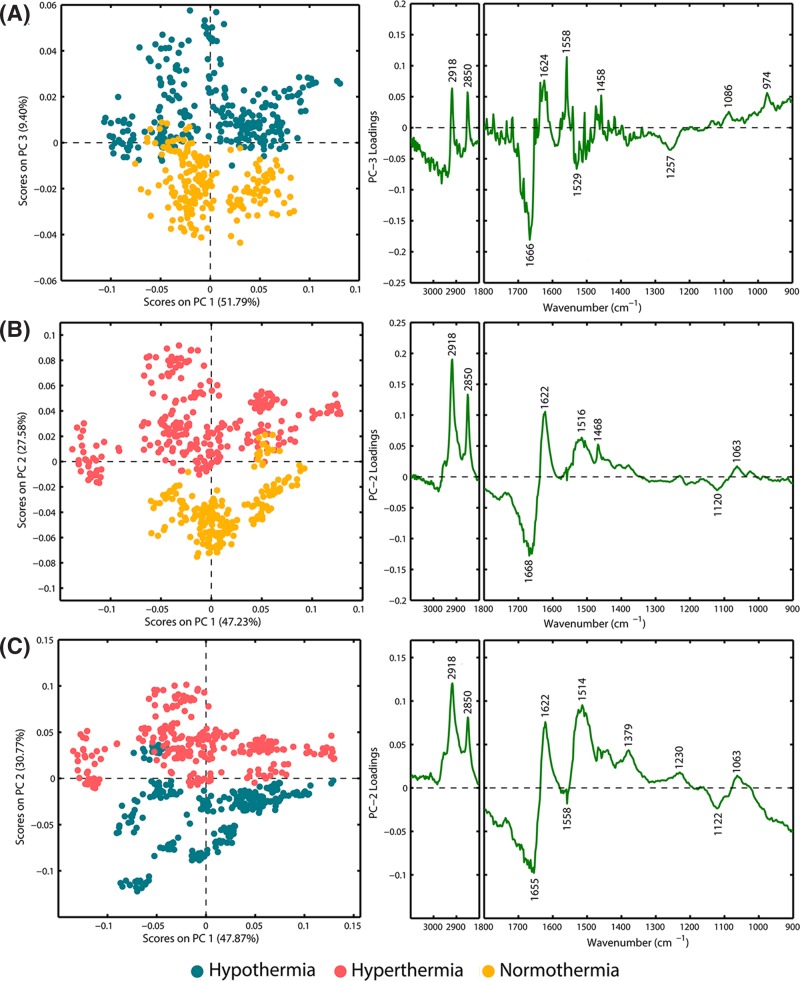
PCA score and loading plots (**A**) PCA results for spectra from fatal hypothermia and normothermia hypothalamus tissues: scores plot (PC-1 vs. PC-3) and loading corresponding to PC-3); (**B**) PCA results for spectra from fatal hyperthermic and normothermic hypothalamus tissues: scores plot (PC-1 vs. PC-2) and loading corresponding to PC-2); (**C**) PCA results for spectra from fatal hypothermic and hyperthermic hypothalamus tissues: scores plot (PC-1 vs. PC-2) and loading corresponding to PC-2).

PCA was employed to differentiate the FTIR-based profiles of hypothalamus tissues in the fatal hypothermia, fatal hyperthermia and normothermia groups. In the first step, for each spectroscopic image, every ten spectra randomly extracted from the corresponding spectral dataset were averaged to represent one spectrum. A spectrum that had been selected once for computing a mean spectrum was not allowed to be selected again for the computation of another mean spectrum. Consequently, 272, 272 and 208 mean spectra, corresponding to the fatal hypothermia, fatal hyperthermia and normothermia groups, respectively, were calculated and then mean-centered for PCA. The PCA results, presented as scores and loadings plots for fatal hypothermia-control, fatal hyperthermia-control and fatal hypothermia-fatal hyperthermia groups are displayed in Figure 3A–C, respectively. As seen in this figure, each score’s plot shows a clear binary separation. The loadings plots that identify new meaningful variables responsible for the discrimination of the groups appearing in the scores plots are also shown in Figure 3A–C. The ‘M’-shape and inverted ‘N’-shape observed in the 3100−2800 cm^−1^ and 1700−1600 cm^−1^ regions of the PC-loadings plot for Figure 3A,B could underline both an increase in the lipid components and an alteration in the secondary conformational structures of the proteins of fatal hypothermia and hyperthermia groups in comparison with the normothermia group. These findings are in accordance with our aforementioned results of the differential spectra analysis. Additionally, multiple positive and negative peaks observed in the 3100−2800 and 1700−900 cm^−1^ regions of the PC-loadings plot for Figure 3C indicate varying concentrations of biomolecules in the fatal hypothermia and hyperthermia groups. This is understandable because the pathophysiology processes of the hypothalamus response to fatal hypothermia and hyperthermia are different. PCA for all the three examined animal groups has been performed (Fig. S2). Fig. S2 indicates that there is a separation trend between these three groups.

### Random forest feature importance analysis

**Figure 4:**
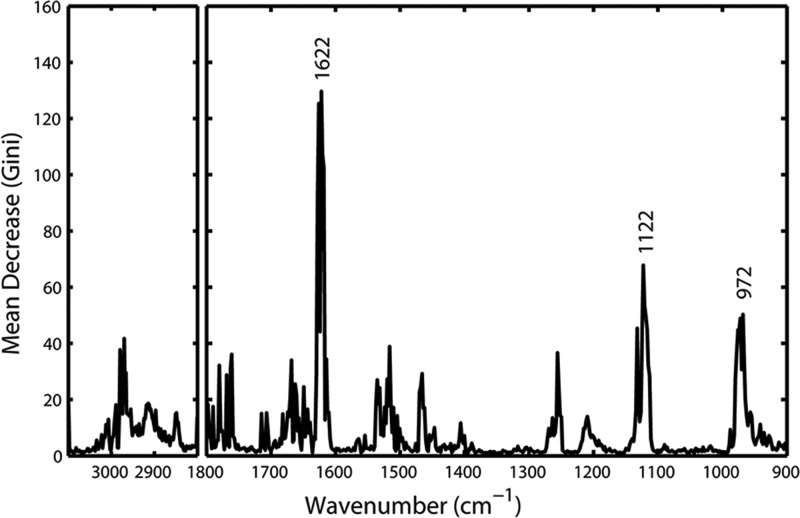
Gini importance chart showing the Mean Decrease in Gini coefficient for all wavenumbers in a range

It has been reported that the random forest classifier with its associated Gini feature importance allows for an explicit feature selection [70]. In this study, we performed RF to discriminate the spectral profiles of the fatal hypothermia, fatal hyperthermia and normothermia groups and further analyze the Mean Decrease Gini coefficient, one important outcome of the RF classification model, to ascertain specific spectral features that are important to the classification. Figure 4 shows the Mean Decrease in Gini Coefficient for all wavenumbers in a range. The higher the score, the more important the wavenumber is to the fatal hypothermia-fatal hyperthermia-normothermia classification model. As can be seen, the prominent wavenumbers in terms of RF importance are around 1622 cm^−1^ (β-sheet secondary structures of proteins) [65], 1122 cm^−1^ (C-O stretching of ribose vibration of RNA) [71] and 972 cm^−1^ (the DNA), suggesting the major differences in spectra seen in proteins and nucleic acids are responsible for the greatest discrimination between fatal hypothermia, fatal hyperthermia and normothermia groups.

### The comparison of the biochemical features among the fatal hypothermic, fatal hyperthermic and normothermic hypothalamus tissues

In this study, we successfully employed three progressive analytical methods, from the basic difference spectra analysis to unsupervised PCA, then to supervised RF machine learning method, to uncover the differences in the spectral profiles of fatal hypothermic, fatal hyperthermic and normothermic hypothalamus tissues. It was found that spectral bands around 2920 and 2850 (lipid feature), 1668, 1626, 1622 and 1518 (protein feature), 1126 (carbohydrate feature) and 1122 and 972 cm^−1^ (nucleic acids feature) were of great value for this discrimination. These findings suggested variations in the mechanisms of lipids, proteins, carbohydrates and nucleic acids across these three groups. To ascertain these mechanism differences and gain further insight into the pathophysiological processes of the hypothalamus response to fatal hypothermia and hyperthermia, these spectral features are discussed more in detail below.

#### Lipid region

**Figure 5:**
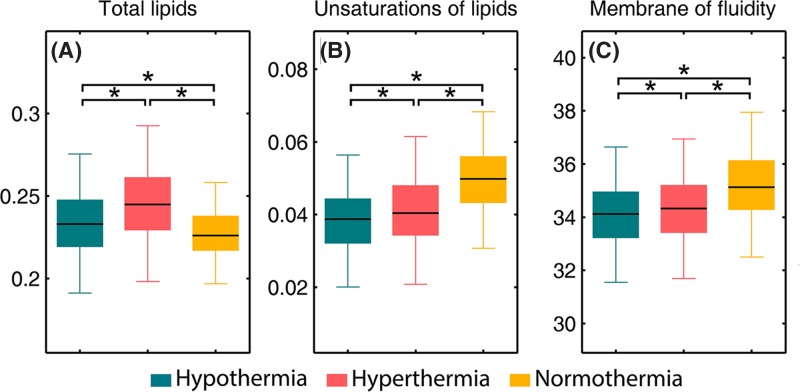
Selected biomarkers found in FTIR spectra (**A**) total lipids (*I*_2850_ + *I*_2920_), (**B**) unsaturation of lipids (*I*_3012_ / (*I*_2850_ + *I*_2920_)), (**C**) membrane fluidity (the bandwidth values of CH_2_ asymmetric stretching band near 2920 cm^−1^). An asterisk means that there is a significant difference (*P*<0.05).

Brain tissue contains the second highest lipid content after adipose tissue and pathological processes could result in alterations both in the content and composition of the brain lipids [72]. Figure 5 presents calculated integral intensities of selected bands and their ratios to illustrate spectral changes of lipids observed in hypothalamus. The sum of the CH_2_ asymmetric and symmetric stretching modes was calculated as the content of total lipids [65] (Figure 5A). This showed the total lipid content to be significantly higher in the fatal hypothermia and hyperthermia groups when compared with normothermia group. It is thought that this is caused by the cerebral ischemia, which often occurs in deaths related to fatal hypothermia and hyperthermia, resulting in an increased production of free fatty acids [73]. The intensity of the olefinic band at 3012 cm^−1^ was used as an index of relative concentration of double bonds in the lipid structure of unsaturated lipids [74,75]. In order to examine the unsaturation level of the hypothalamus tissues, olefinic C-H/ total lipid ratio was calculated. Decreases in the olefinic C-H/ total lipid ratio both in the fatal hypothermia and hyperthermia groups with respect to the normothermia group (Figure 5B) demonstrate decreases in the concentration of unsaturated fatty acids in these two groups. These results could be attributed to lipid peroxidation, which causes the loss of olefinic bonds by breaking double bond sites of polyunsaturated acyl chains [74]. The production of free radicals induced by cell injury is known as an important pathogenesis for lipid peroxidation [76]. Therefore, we speculate that the neurocyte injuries of hypothalamus tissues induced by fatal hypothermia and hyperthermia could produce free radicals and further result in lipid peroxidation. Additionally, the bandwidths of CH_2_ asymmetric stretching bands were also measured for half of the peaks in this study. According to previous literature, these bandwidths are related to the motional rates of the molecule and are evidence of the membrane dynamics [77,78]. Significant decreases were observed in the bandwidth values of CH_2_ asymmetric stretching bands of fatal hypothermia and hyperthermia groups when compared with the control group (Figure 5C), indicative of lower membrane fluidity of neurons in fatal hypothermic and hyperthermic hypothalamus tissues.

#### Protein region

**Figure 6:**
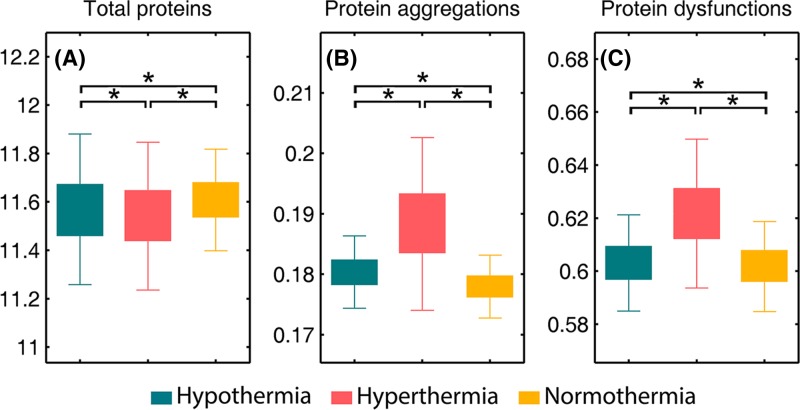
Selected biomarkers found in FTIR spectra (**A**) total proteins (∑(*I*_1600_- *I*_1700_)), (**B**) protein aggregates (*I*_1622_), (**C**) protein dysfunction (*I*_1545_ / *I*_1655_). An asterisk means that there is a significant difference (*P*<0.05).

The FTIR-based amide profiles have proven to be valuable for examining the content, structure and function of tissue proteins [67,79]. Here, the sum of intensities of protein amide I band (1700−1600 cm^−1^), which corresponds mainly to the C = O stretching modes of the protein backbone [80], were calculated to illustrate the total protein level (Figure 6A), while the intensity of 1622 cm^−1^ was calculated to represent the level of aggregated proteins (Figure 6B) [52,53]. Interestingly, it is observed that the fatal hyperthermia group harbors both the lowest content of total proteins and the highest content of protein aggregates among these three groups. The decreased level of total proteins could be caused by a down-regulation of proteins in the neurocyte response to heat stress [81], and the increased level of protein aggregates could be attributed to brain ischemia, which causes energy deprivation, free radical generation and antioxidant depletion as well as an increase of tissue oxidation products like protein aggregates [53]. In this study, we also calculated the ratio of amide II (maximum peak at 1545 cm^−1^) to amide I (maximum peak at 1655 cm^−1^), a well-known spectral marker of protein secondary structure alteration induced by various stressors [67,82,83], to represent the level of dysfunctional proteins. The graph in Figure 6C shows the highest value of the amide II/amide I ratio occuring in the fatal hyperthermia group, indicative of the most severe protein dysfunction level in fatal hyperthermic hypothalamus tissues. The three graphs in Figure 6 together demonstrate that the content, structure and function of proteins, and the cellular activity of hypothalamus tissues, are more susceptible to hyperthermia than hypothermia. Indeed, some authors point out that despite the damaging effects of hypothermia, body cooling can substantially increase the ischemic tolerance of the internal organs [84]. Our experimental data revealed a longer average death time for rats in the fatal hypothermia group than in the fatal hyperthermia group, which indirectly supports that hypothesis.

#### Carbohydrate and nucleic acid region

**Figure 7:**
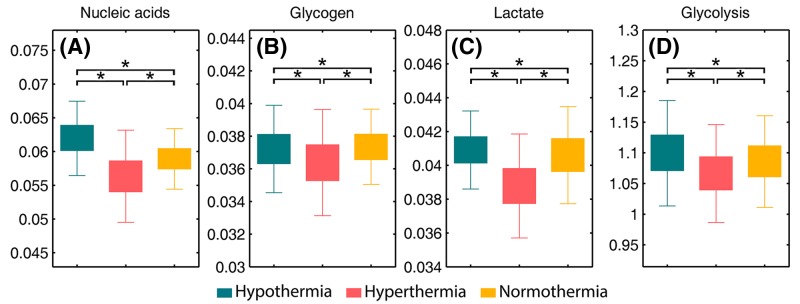
Selected biomarkers found in FTIR spectra (**A**) total nucleic acids (*I*_1122_ + *I*_972_), (**B**) glycogen (*I*_1151_), (**C**) lactate (*I*_1126_), (**D**) glycolysis (*I*_1126_ / *I*_1151_). An asterisk means that there is a significant difference (*P*<0.05).

The sum of intensity at 1122 (arising from RNA) and 972 cm^−1^ (arising from DNA) illustrating the total nucleic acid levels was calculated (Figure 7A). As can be observed, the total nucleic acid content is significantly lower in the fatal hyperthermia group compared with normothermia group, which is thought to be a result of heat stress. At a cellular level, this stress induces inhibition of DNA synthesis and transcription, RNA splicing and translation and cell-cycle inhibition [85,86]. A small but significant increase in the nucleic acid content for the fatal hypothermia group with respect to normothermia group is also found. This finding is in accordance with previous literature, which reported that mild hypothermia may enhance the expression of neuroprotective or repair genes to protect nervous tissues [87].

FTIR has also proved valuable for investigating carbohydrates metabolic pathways in the brain [51]. In this study, we performed absorption integration from the most specific IR bands of glycogen (1151 cm^−1^) [51] (Figure 7B) and its one metabolic product, lactate (1126 cm^−1^) (Figure 7C). The ratio of lactate to glycogen (1126 cm^−1^/1151 cm^−1^) was also calculated to evaluate glycolysis progress (Figure 7D). We observed that the fatal hyperthermia group contained a lower content in both glycogen and lactate in comparison with the normothermia group. This is understandable as the raised body temperature during the early pathological process of hyperthermia increases the metabolic rate and oxygen consumption, resulting in the consumption of carbohydrates [88]. Additionally, Figure 7D shows that the fatal hypothermia group has a more active glycolysis process than the hyperthermia group. It is reported that a prolonged ischemic condition could lead to the survival of cells (i.e. glia and macrophages) only capable of glycolysis metabolism [51]. Taken together, our experimental results reveal a longer average death time for rats in the fatal hypothermic tissue group than those of the fatal hyperthermic group. Previous literature has reported that heat stress not only directly affects the central nervous system and induces cytotoxicity, which kills cells, but also indirectly induces blood redistribution toward skin and muscles, resulting in neuronal necrosis [81,88]. We speculate that the fatal hypothermia group underwent a longer period of ischemia than the fatal hyperthermia group, which led to the more active glycolysis seen in the fatal hypothermia group.

## Conclusion

In this preliminary study, we demonstrate for the first time the potential of FTIR spectroscopy combined with advanced chemometrics as a method for easy and rapid assessment of the biomolecular variations seen in fatal hypothermic, fatal hyperthermic and normothermic hypothalamus tissues in rat models. Our FTIR-based results showed that the fatal hypothermic and hyperthermic hypothalamus tissues both have higher total lipid levels and lower lipid unsaturation levels, and their cell membranes demonstrated less motional freedom in comparison with the normothermic hypothalamus tissue. Additionally, the lowest total protein and carbohydrate levels and the highest protein aggregates and dysfunctions levels were observed in the fatal hyperthermic hypothalamus tissues, while the highest nucleic acid and glycolysis levels were observed in the hypothermic hypothalamus tissues.

The brain often remains unexamined in fatal hypothermia/hyperthermia death investigations because it is thought to only indicate nonspecific histopathological findings. Nevertheless, our FTIR-based results show that the biochemical profiles of fatal hypothermic, fatal hyperthermic and normothermic hypothalamus tissues were significantly different. This study, supported by our previous studies [89,90], demonstrates that some tissues or organs regarded as nonspecific for the determination of the cause of death in forensic pathology do indeed have specific biochemical fingerprints. There is a high potential for this technique to augment the results of conventional forensic post-mortem examination and provide for a more accurate determination of the cause of death. Nevertheless, much work still needs to be done both by our own group and by others. It is important to acknowledge that we need to investigate the biochemical signatures of hypothalamus tissues from alternative causes of death, such as fatal anaphylactic shock, drowning, sudden cardiac death, neurological failures and intoxication-related deaths. These are common causes of death encountered in forensic casework and should be taken into consideration in the control groups of further studies like this one.

## Supporting information

**Figure S1.** (A) The inferior view of the rat’s brain. (B) the medial view of a saggital section of the rat’s brain. To make sure the anatomical structures of hypothalamus regions that selected for infrared measurement from all the rat donors are as consistent as possible, the brain coronal sections of same position from all rat donors were cut. The blue lines in the figure illustrate the position that the brain coronal sections were cut. The position is in front of pituitary stalk.

**Figure S2.** PCA results for spectra from fatal hypothermia, fatal hyperthermia and normothermia hypothalamus tissues: (A) scores plot (PC-1 vs. PC-2 vs. PC-3) and (B) loading corresponding to PC-1, PC-2 and PC-3. The separation trend between these three groups is observed.

## Abbreviations

EMSC: extended multiplicative signal correction
FTIR: fourier-transform infrared
H&E: hematoxylin & eosin
PCA: principle component analysis
RF: random forest
