# Supplementary material for: Biochemical detection of fatal hypothermia and hyperthermia in affected rat hypothalamus tissues by Fourier transform infrared spectroscopy
Source: Biosci Rep. 2019 Mar 15;39(3):BSR20181633. doi: 10.1042/BSR20181633 (PMC6418404; doi:10.1042/BSR20181633)
Supplement: Supplementary file 1 [file bsr-39-bsr20181633_Supp1.pdf]

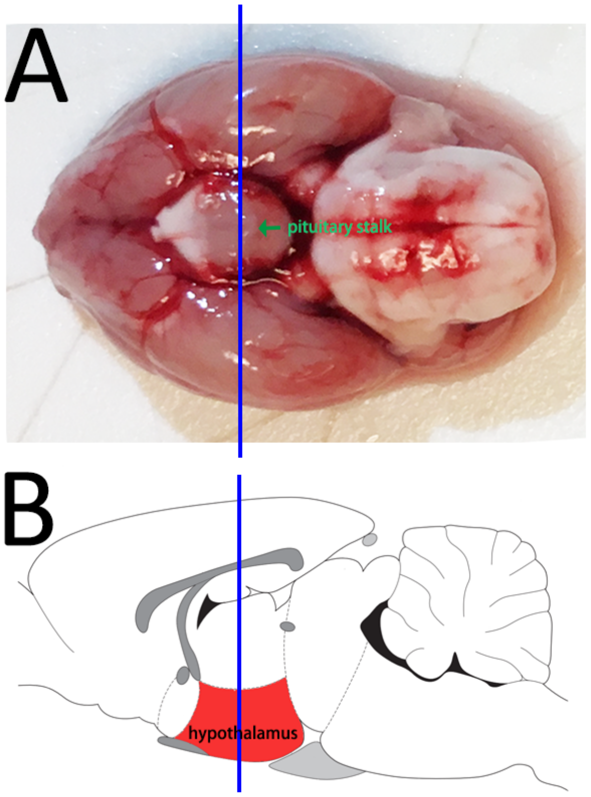

Figure S1. (A) The inferior view of the rat's brain. (B) the medial view of a saggital section of the rat's brain. To make sure the anatomical structures of hypothalamus regions that selected for infrared measurement from all the rat donors are as consistent as possible, the brain coronal sections of same position from all rat donors were cut. The blue lines in the figure illustrate the position that the brain coronal sections were cut. The position is in front of pituitary stalk.

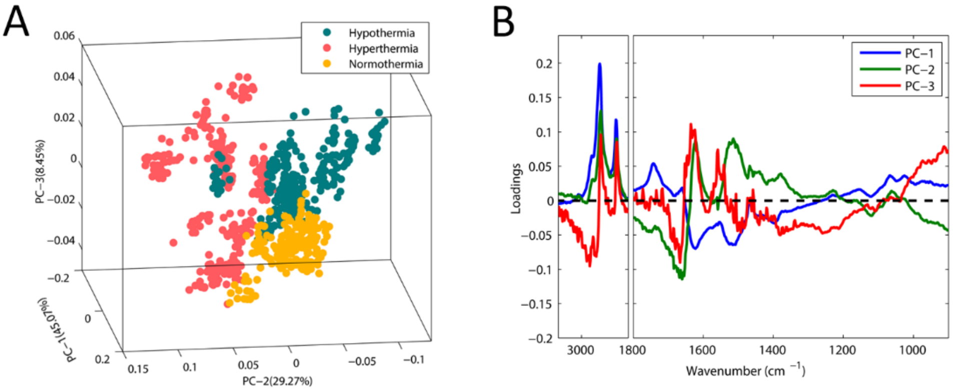

Figure S2. PCA results for spectra from fatal hypothermia, fatal hyperthermia and normothermia hypothalamus tissues: (A) scores plot (PC-1 vs. PC-2 vs. PC-3) and (B) loading corresponding to PC-1, PC-2 and PC-3. The separation trend between these three groups is observed.
